# Supplementary material for: K12-ligand-based CAR T cell therapy for CD7-positive T cell malignancies
Source: Mol Ther Oncol. 2025 Apr 29;33(2):200988. doi: 10.1016/j.omton.2025.200988 (PMC12208613; doi:10.1016/j.omton.2025.200988)
Supplement: Document S1. Figures S1–S3 [file mmc1.pdf]

OMTON, Volume *33*

Supplemental information

K12-ligand-based CAR T cell therapy for CD7-positive T cell malignancies

Nienke Visser, Macarena González-Corrales, Jimena Álvarez-Freile, Maurien G. Pruis, Lena Rockstein, Harm Jan Lourens, Jan Jacob Schuringa, Tom van Meerten, Gerwin Huls, and Edwin Bremer

Figure S1

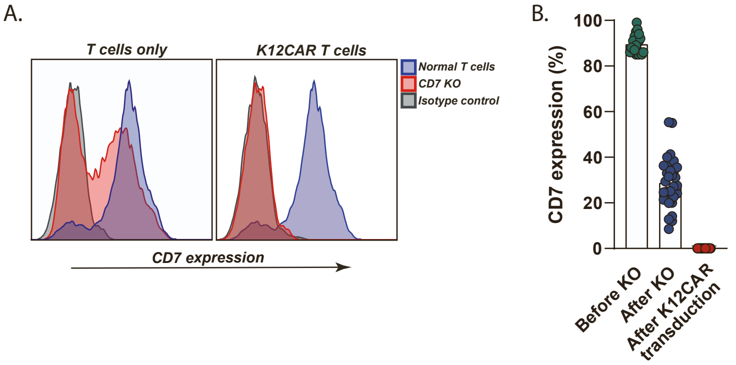

Figure S1 **Fratricidal effect of K12 CAR towards CD7-positive T cells.**
(A-B) CD7 expression in T cells before and after CD7KO and K12 CAR transduction

Figure S2

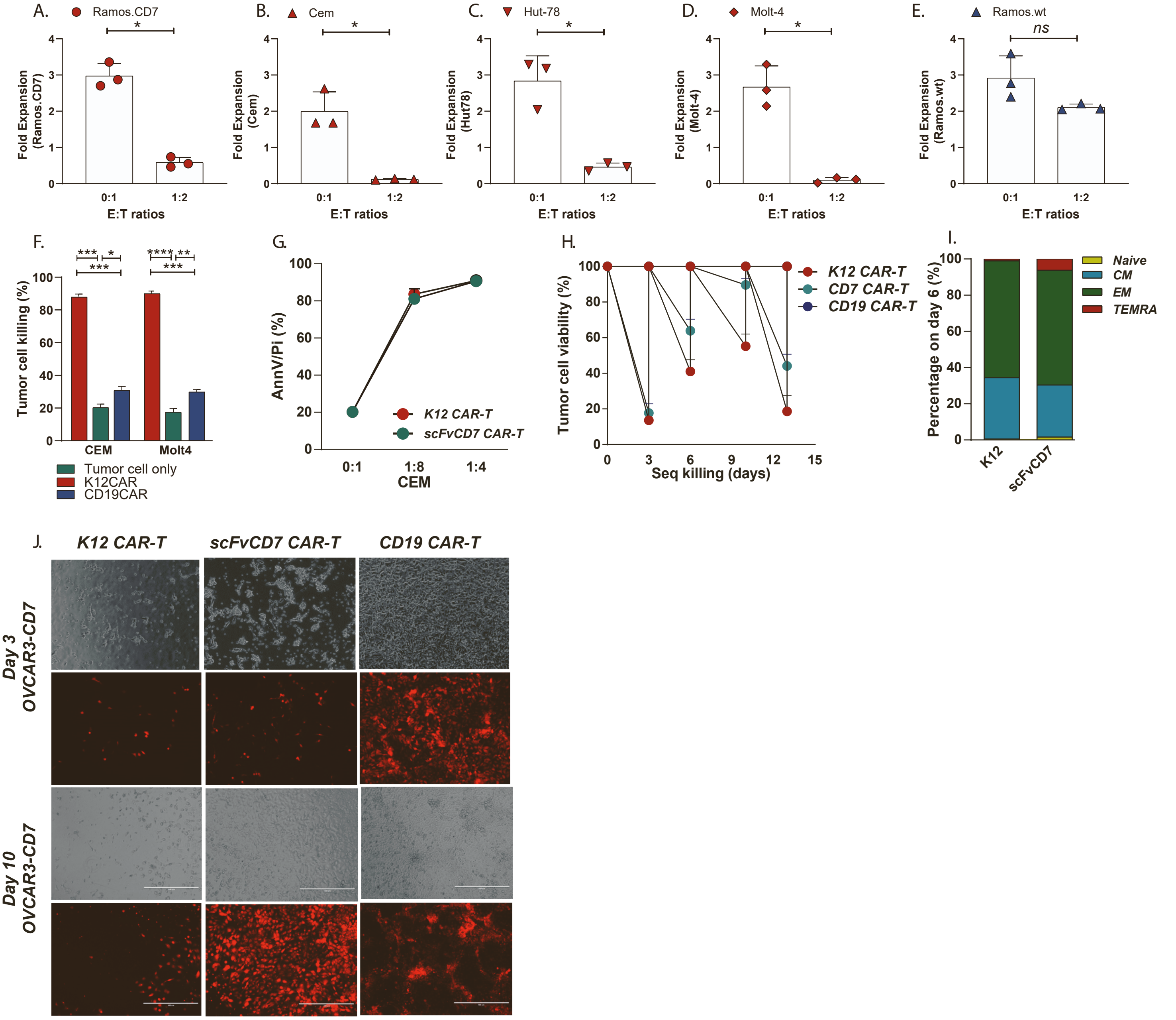

Figure S2 **K12 CAR-T cells selectively eliminated CD7-positive tumor cells.** Remaining tumor cells quantified as Annexin V/PI- negative cells, after treatment with K12 CAR-T cells, for 48h. **(A)** Ramos.CD7, **(B)** CEM, **(C)** Hut-78, **(D)** Molt-4**,** **(E)** Ramos.wt. **(F)** Cell death of CEM and Molt-4 treated for 48h with K12 CAR or CD19 CAR-T cells. **(G)** 24h killing assay with CEM tumor cell lines, with scFvCD7 CAR-T cell and K12 CAR-T cells. Upon various E:T ratios. **(H)** Sequential killing with OVCAR-3.CD7 and K12, scFv7 and CD19 CAR-T cells. **(I)** T cell subsets of scFvCD7 and K12 CAR T cells after 6 days of manufacturing. **(J)**. Bright-field microscopy pictures of OvCAR-3.CD7-mCherry in co-culture with K12 CAR-T cells, scFvCD7 CAR-T cells and CD19 CAR-T cells, on day 3 and day 10 of the repeated killing assay.
p values are: ****p < 0.0001, ***p < 0.001, **p < 0.01, and *p < 0.05.

Figure S3

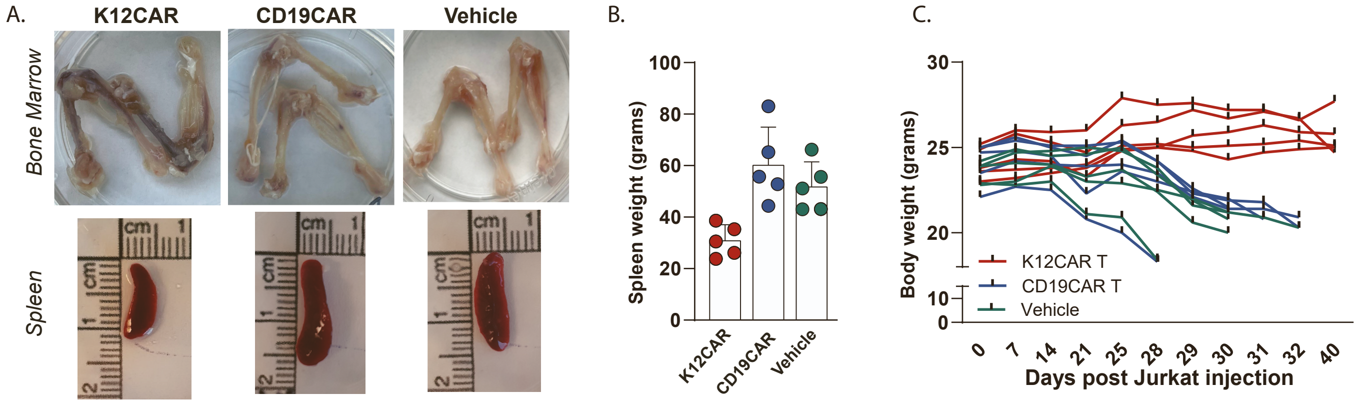

Figure S3 **K12 CAR-T cells eliminated Jurkat cells in a mouse xenograft model**
(A) Representative images of bone marrow and spleen collected post-sacrifice from mice treated with K12, CD19 CAR-T cells, or vehicle control. **(B)** Spleen weight measured post-sacrifice from mice treated with K12, CD19 CAR-T cells, or vehicle control. **(C)** Body weight in grams of the mice treated with K12, CD19 CAR-T cells or vehicle control.
