# Supplementary material for: K12-ligand-based CAR T cell therapy for CD7-positive T cell malignancies
Source: Mol Ther Oncol. 2025 Apr 29;33(2):200988. doi: 10.1016/j.omton.2025.200988 (PMC12208613; doi:10.1016/j.omton.2025.200988)
Supplement: Document S2. Article plus supplemental information [file mmc2.pdf]

Molecular Therapy
Oncology
Original Article

K12-ligand-based CAR T cell therapy for CD7-positive T cell malignancies

Nienke Visser,[1,2] Macarena González-Corrales,[1,2] Jimena Álvarez-Freile,[1] Maurien G. Pruis,[1] Lena Rockstein,[1] Harm Jan Lourens,[1] Jan Jacob Schuringa,[1] Tom van Meerten,[1] Gerwin Huls,[1] and Edwin Bremer[1]

[1]Department of Hematology, University Medical Center Groningen, University of Groningen, 9713 GZ Groningen, the Netherlands

Chimeric antigen receptor (CAR)-based therapy is of interest for relapsed or refractory (r/r) T cell acute lymphoblastic leukemia (T-ALL) and T cell lymphomas. A prominent target antigen for this is the receptor CD7, which is expressed in ∼95% of T-ALL, ∼50% of peripheral T cell lymphomas, as well as 10% of acute myeloid leukemias. Here, we preclinically evaluated and compared CD7-targeted ligand K12-based CAR-T to an scFvCD7-based CAR-T construct. K12 CAR-T cells produced significantly higher interferon gamma (IFN-γ) after CD7 activation compared to scFv-CD7 CAR-T cells. Similarly, in a Jurkat$^{NFAT\text{-}luc}$ reporter cell line expressing the respective CAR, CD7-induced luminescence was significantly higher by the K12 CAR than the scFv-CD7 CAR. K12 CAR-T treatment selectively and specifically eliminated a panel of CD7-positive, but not CD7-negative, cell lines and eliminated acute-T-cell-leukemia-patient-derived and acute myeloid leukemia blasts in an effector-to-target ratio-dependent manner. Further, K12 CAR-T cells had prominent anti-leukemic activity in an intravenously (i.v.) injected Jurkat leukemia mouse model, with no detectable disease in three out of five mice treated with K12 CAR-T. Therefore, K12 CAR T cell therapy might be of use for the treatment of r/r patients with CD7-positive T cell leukemia/lymphoma and acute myeloid leukemia (AML).

INTRODUCTION

T cell acute lymphoblastic leukemia (T-ALL) and T cell lymphomas are highly aggressive hematological malignancies, with a 5-year overall survival (OS) rate lower than 20% for patients with relapsed or refractory (r/r) disease.[1–3] Thus, new therapeutic approaches are urgently needed for these malignancies. In this respect, chimeric antigen receptor (CAR) T cell therapy holds considerable promise. CAR T cell therapy for T cell malignancies is less advanced than for B cell malignancies, but various clinical trials have been reported with T-cell-targeted CAR-Ts against, among others, CD5,[3–5] CD30,[6,7] CD99,[8] and CD7.[9–12]

For T cell malignancies, the 40 kDa transmembrane glycoprotein CD7 is a particularly interesting target as it is expressed in ∼95% of T-ALL and ∼50% of peripheral T cell lymphomas.[1,13,14] CD7 is also expressed in the majority of normal T cells.[15] The function of CD7 is as yet incompletely characterized, but binding of CD7 by its ligand K12/SECTM1 provided co-stimulatory signaling and played a role in T cell activation.[16] However, disruption of CD7 in murine T cells did not alter T cell development or homeostasis, indicating CD7 is not critical for development.[16] Furthermore, ∼5%–15% of healthy T cells lack CD7, with these CD7-negative T cells having a predominant CD4+ memory phenotype (CD45RO+/CD45RA-).[17] Notably, no differences in expansion, exhaustion, or anticancer cytotoxicity were identified when CD19 CAR-T cells were generated from CD7-negative or bulk T cells.[18] Thus, T cells lacking CD7 were functionally indistinguishable from bulk T cells in terms of CAR-T cell activity preclinically.

Since CD7 is an antigen shared between healthy and malignant T cells, fratricidal effects toward "healthy" CAR-T cells need to be prevented. Various options have been reported for this purpose, including knockout of CD7 using clustered regularly interspaced short palindromic repeats (CRISPR)/Cas9 gene editing,[16,19] selection and transduction of CD7-negative T cells,[18] use of a protein expression blocker (PEBL) against CD7,[20] or use of a natural selection method for CD7 downregulation,[12] with all of these strategies having been preclinically validated.

The targeting of the antigen CD7 with anti-CD7 CAR-Ts has been evaluated in various clinical trials for T-ALL or T cell lymphoma patients.[12,19–21] Treatment with third-generation CD7 CAR-T cells yielded complete remission (CR) in 19/20 patients and an 18-month OS rate of 75%.[12] Second-generation allogeneic anti-CD7 CAR-T cells yielded a 28-day CR rate of ∼64%.[1] In another study, with a second-generation allogeneic CAR, durable responses of 85% CR were reported in a subset of r/r T-ALL with a median follow-up time of 27 months (17/20 patients),[22] and a recent phase I trial with sequential allogeneic CD7 CAR T cell therapy and haploidentical stem cell transplantation resulted in an estimated 1-year OS of 68%.[23] Taken together, CD7-targeted CAR T cell therapy for

Received 20 December 2024; accepted 25 April 2025;
https://doi.org/10.1016/j.omton.2025.200988.

[2]These authors contributed equally

Correspondence: Prof. Dr. E. Bremer, Department of Hematology, University Medical Center Groningen, University of Groningen, 9713 GZ Groningen, the Netherlands.
E-mail: e.bremer@umcg.nl

© 2025 The Author(s).
Published by Elsevier Inc. on behalf of The American Society of Gene and Cell Therapy.
This is an open access article under the CC BY license (http://creativecommons.org/licenses/by/4.0/).

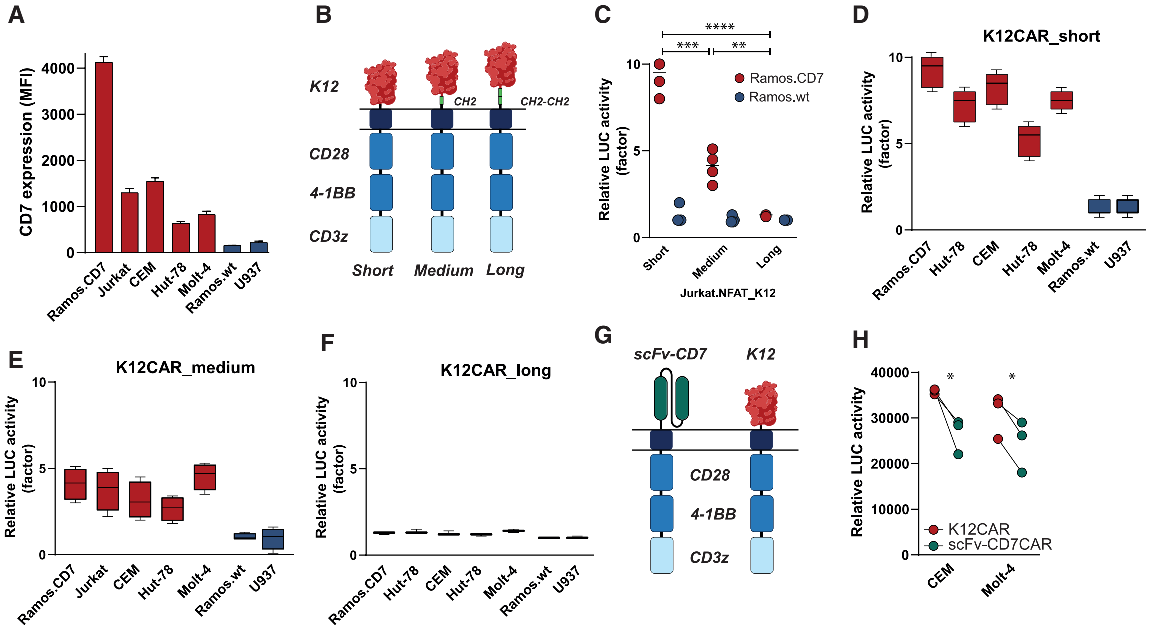

Figure 1. CD7-positive tumor cells selectively activated Jurkat$^{NFAT-luc}$K12 CAR to a higher extent than Jurkat$^{NFAT-luc}$scFv-CD7 CAR
(A) Expression of CD7 on various CD7-positive and CD7-negative leukemic cell lines as mean fluorescent intensity (MFI). (B) Schematic illustration of K12 CAR short, medium, and long constructs. (C) Relative luminescence units (RLU) measured after 24 h co-culture of Jurkat$^{NFAT-luc}$K12 CAR (short, medium, and long isoform) as a fold change compared to control untreated cells. (D) RLU measured after 24 h co-culture of a panel of CD7-positive and negative cell lines with Jurkat$^{NFAT-luc}$K12 CAR short isoform, (E) medium isoform, and (F) long isoform. (G) Schematic illustration of scFvCD7 and K12 CAR-T cell constructs. (H) RLU generated by scFvCD7 and K12 CAR Jurkat$^{NFAT-luc}$ cells after co-culture with CEM and Molt-4. ****$p < 0.0001$, ***$p < 0.001$, **$p < 0.01$, and *$p < 0.05$.

T-ALL and T cell lymphoma is safe and has shown promising signs of efficacy.

So far, the clinically evaluated CD7-targeting CAR-T cells have predominantly used prototypical antibody derivatives of the so-called single-chain fragments of variable regions (scFv) format. However, several studies have demonstrated that instability of the scFv can lead to oligomerization and domain swapping where the V_H region of one scFv will incorrectly bind with another V_L region from a different scFv domain.[24,25] This feature can result in scFv CAR aggregates and trigger tonic signaling, which promotes T cell exhaustion and adversely affects *in vivo* efficacy.[21,26] To circumvent this issue, natural ligands can be exploited as targeting moiety. This is particularly appealing for CD7 as its ligand K12 (SECTM1) exhibits a relatively high affinity, with separate reports indicating values of 10 and 38 nM,[27,28] which is reported to be within the optimal range for CAR affinities.[29,30] We previously successfully exploited K12 for therapeutic targeting of CD7 with an immunocytokine,[31] and a second-generation K12/SECTM1-based CAR-T cell with prominent CD7-restricted anticancer activity was recently reported.[32] However, comparative clinical outcomes of third-generation versus second-generation CD19 CAR-T cells in targeting B cell non-Hodgkin lymphoma indicate superior persistence and up to 40-fold greater expansion for the third-generation CD19 CAR-T cells.[33] Further, as described earlier, the third-generation anti-CD7 CAR yielded a very high initial CR rate of 95%, indicating therapeutic promise of this CAR design.[12] Therefore, in this study, we preclinically evaluated a third-generation K12-ligand-based CAR-T and identified that CD7-edited (CD7KO) K12 CAR-T cells produced significantly more interferon gamma (IFN-γ) after CD7-specific activation than scFvCD7-based CAR-T cells. Similarly, in a Jurkat$^{NFAT-luc}$ reporter cell line expressing the respective CAR, CD7-specific triggering of CAR signaling yielded significantly higher luminescence by the K12-CAR than the scFvCD7-CAR. Further, K12 CAR-T cells selectively eliminated CD7-positive T-ALL cell lines and primary leukemia blasts *in vitro* and had prominent tumoricidal activity in mice, with three out of five mice having no detectable disease. Taken together, these results support the feasibility of targeting T-ALL with K12-ligand-based CAR-T cells as a viable alternative to scFv-CD7-based CAR-T.

RESULTS

CD7-positive tumor cells activated Jurkat$^{NFAT-luc}$K12 CAR to a higher extent than Jurkat$^{NFAT-luc}$scFvCD7 CAR

To investigate CD7-restricted activity of K12 and scFv-CD7 CAR-T cells, a panel of cell lines with a broad range of CD7 expression was included, varying from negative (Ramos.wt and U937), endogenously expressing CD7 (Hut-78, Molt-4, Jurkat, CEM) to ectopically and very highly expressing CD7 (Ramos.CD7) (Figure 1A). To delineate the optimal configuration of the extracellular recognition domain for a K12-based CAR, three different CAR variants with varying extracellular domain lengths were constructed with no,

one, or two IgG2 CH2 spacer domains (Figure 1B). Upon CAR transduction of these variants into Jurkat$^{NFAT\text{-}luc}$ cells, a model system to analyze NFAT-driven T cell activation using luminescence, co-culture of Jurkat$^{NFAT\text{-}luc}$.K12 CAR^{short} and Jurkat$^{NFAT\text{-}luc}$.K12 CAR^{medium} with Ramos.CD7 significantly upregulated luminescence compared to monocultures of reporter cell lines (Figure 1C). In contrast, co-cultures of Jurkat$^{NFAT\text{-}luc}$.K12 CAR^{long} did not significantly induce luminescence. Notably, induction of luminescence by K12 CAR^{short} was significantly higher than by K12 CAR^{medium} (Figure 1C, $p < 0.001$). Importantly, the percentage of luminescence induction in co-cultures with Ramos.wt was only ∼10% of the Ramos.CD7 signal for K12 CAR^{short} and K12 CAR^{medium}, with luminescence levels comparable to single K12 CAR reporter cultures. Thus, both K12 CAR^{short} and K12 CAR^{medium} did not display tonic signaling in this model system. Similar co-culture of these reporter lines with a cell line panel revealed that Jurkat$^{NFAT\text{-}luc}$.K12 CAR^{short} luminescence was 5- to 10-fold increased in co-cultures with endogenous CD7-positive cells compared to CD7-negative cells (Figure 1D). Co-cultures with Jurkat$^{NFAT\text{-}luc}$.K12 CAR^{medium} yielded a CD7-specific increase in luminescence between 2- and 5-fold (Figure 1E), whereas Jurkat$^{NFAT\text{-}luc}$.K12 CAR^{long} failed to upregulate luminescence (Figure 1F). As the K12 CAR^{short} yielded the highest CD7-specific response in the absence of tonic signaling, the K12 CAR^{short} (hereafter termed K12 CAR) was further evaluated throughout this manuscript.

Next, the ligand-based K12 CAR construct was evaluated in a comparative study with an analogous scFvCD7-based CAR comprising antibody fragment scFv3A1F (Figure 1G), an scFv used previously in a pre-clinical anti-CD7 CAR study.[34] Of note, as various scFvCD7-based CAR-T cells using the "short" extracellular domain have already been reported,[35] the comparative study was performed with the scFvCD7^{short} CAR construct, using the Jurkat$^{NFAT\text{-}luc}$ system. Interestingly, the increase in luminescence obtained from Jurkat$^{NFAT\text{-}luc}$.K12 CAR was significantly higher than with Jurkat$^{NFAT\text{-}luc}$.scFvCD7 CAR upon co-culture with CEM (RLU increase of 3.6×10^4 vs. 2.7×10^4, $p < 0.05$) and Molt-4 (RLU increase of 3.2×10^4 vs. 2.3×10^4, $p < 0.05$) (Figure 1H). Taken together, the K12 CAR was characterized by CD7-specific CAR activity that was significantly higher than scFvCD7 CAR in the Jurkat$^{NFAT\text{-}luc}$ model system.

K12 CAR-T cells outperformed IFN-γ secretion of scFvCD7 CAR-T cells and were highly cytotoxic against CD7-positive T-ALL cells

The therapeutic potential of CD7-targeted CAR-T cells was further evaluated using primary T cells. Prior to CAR transduction, CD7 expression was removed using CRISPR/Cas9, as CD7 is stably expressed on healthy mature T cells and CAR transduction may yield unwanted fratricidal elimination of cells. Knockout efficiency of this procedure was ∼70% (Figure S1A), whereupon K12 or scFvCD7 CAR constructs were transduced (Figure S1A). CAR transduction yielded a further reduction in CD7-positive cells to <5% after 3 days of expansion due to fratricidal elimination (Figure S1B). To delineate the effect of CD7 triggering, K12 and scFvCD7 CAR-T cells that both were >90% CAR-positive (Figure 2A) were stimulated with CD7-loaded dynabeads for 24 h, yielding IFN-γ secretion of ∼1,000 pg/mL by K12 CAR-T cells. This level was significantly higher than the ∼200 pg/mL secreted by scFvCD7 CAR-T cells (Figure 2B). Again, neither K12 CAR-T nor scFvCD7 CAR-T displayed any tonic signaling, with no significant secretion of IFN-γ upon incubation with control CD19-loaded dynabeads (Figure 2B). The opposite effect was observed with CD19 CAR-T cells, where no IFN-γ was detected upon incubation with CD7-loaded beads but ∼1,000 pg/mL was detected upon incubation with CD19-loaded beads (Figure 2B). Thus, K12-CAR T cells responded more efficiently than scFvCD7 CAR-T cells to CD7-specific stimulation.

In subsequent co-cultures, K12 CAR-T cells strongly reduced viability of CD7-positive Jurkat leukemic cells, to ∼2% at effector-to-target (E:T) of 1:1, as demonstrated in representative images derived from flow cytometry (Figure 2C). This reduction was observed in an E:T-ratio-dependent manner in various CD7-positive cell lines, but not the CD7-negative cell line U937 (Figures 2C and 2D), with a mean tumor cell lysis of 74.5% for Ramos.CD7, 69.3% for CEM, 77.8% for Hut78, and 64.0% for Molt-4 (Figure 2D). In line with this, almost no residual CD7-positive cancer cells were detected after co-culture with K12 CAR-T cells, whereas the number of viable CD7-negative cancer cells was not affected compared to single cancer cell cultures (Figures 2E and S2A–S2E). Further, control treatment with CD19 CAR-T cells only marginally triggered cell death compared to non-treated cancer cells (Figure S2F). Thus, K12 CAR-T cells had prominent CD7-restricted cytotoxic activity, also in a sequential killing assay in which cytotoxicity persisted toward CD7-positive tumor cell lines (CEM, Molt-4) for at least six rounds (Figures 2F and 2G). Notably, when comparing K12 CAR-T cell cytotoxicity toward CD7-positive tumor cells, it was found to be comparable to that of scFvCD7 CAR T cells in a first cytotoxicity assay (Figure S2G). However, repeated stimulation of model adherent cell line OVCAR-3.luciferase, ectopically expressing CD7 and fluorescent marker mCherry, yielded a clearly more prominent and persistent cytotoxic effect of K12 CAR-T cells than scFvCD7 CAR T cells as visualized by fluorescent microscopy and quantified by luminescence (Figures S2G and S2H). Interestingly, on the last day of manufacturing of the CAR-T cells (day 6), K12 CAR-T cells had a higher prevalence of central memory (CM) T cells of 34% compared to 28% scFvCD7 CAR. Conversely, scFvCD7 CAR-T cells had a small increase in terminally differentiated T cells (TEMRA cells) of 6% versus 1% (Figure S2H). Further, treatment of CD7-positive cancer cells with K12 CAR-T also triggered robust secretion of IFN-γ (Figure 2H), with a spread in concentration from 6.1–22.5 ng/mL that correlated strongly with CD7 expression on cancer cells (Figure 2I, $R^2 = 0.859$). Taken together, K12 CAR-T cells had prominent cytotoxicity toward CD7-positive tumor cell lines and had increased CD7-restricted activation and prolonged cytotoxic activity compared to scFvCD7 CAR-T cells *in vitro*. As K12 CAR-T cells outperformed scFvCD7 CAR-T cells, a further in-depth analysis for anti-leukemic activity was focused on K12 CAR-T cells.

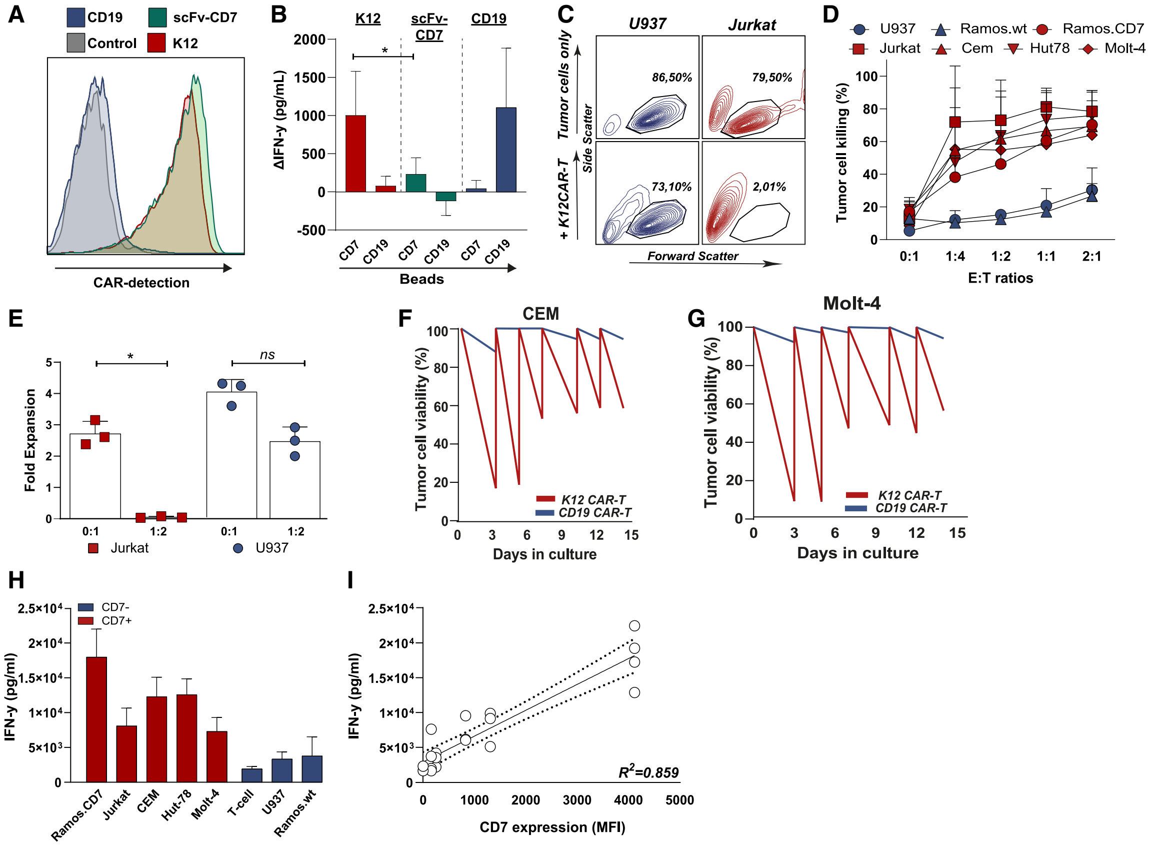

Figure 2. K12 CAR-T cells outperformed scFv-CD7 in terms of IFN-γ

(A) CAR detection on K12 and scFvCD7 CAR-T cells measured by binding of recombinant CD7. (B) IFN-γ secretion after stimulation of K12, CD19, and scFvCD7 CAR-T cells with unloaded, recombinant CD7- or recombinant CD19-loaded beads. (C) Illustrative flow cytometry plots of (CTV-negative) Jurkat and U937 after treatment with K12 CAR-T cells. (D) Quantification of cell death in a panel of T-ALL cell lines at various effector-to-target (E:T) ratios after 48 h treatment with K12 CAR-T cells. (E) Counts of residual viable (CTV-negative) Jurkat and U937, quantified as Annexin V/PI-negative cells (gate based on non-treated tumor cells) after 48 h treatment with K12 CAR-T cells. (F) Quantification of cancer cell viability during six rounds of repeat treatment with K12 CAR-T cells or CD19 CAR-T cells against CEM, and (G) against Molt-4. (H) IFN-γ secretion by K12 CAR-T cells after treatment of tumor cell lines for 48 h at E:T ratio of 1:2. (I) Correlation between CD7 expression in targeted cells (MFI) and IFN-γ secretion (pg/mL), $R^2 = 0.859$. *$p < 0.05$.

K12 CAR-T cells were highly cytotoxic against patient-derived leukemia cells

To further evaluate cytotoxicity of K12 CAR-T cells, both patient-derived T-ALL and acute myeloid leukemia (AML) cells were isolated, with CD7 expression detected in ∼95% of T-ALL blasts (Figure 3A, $n = 7$), a finding in line with literature.[36] In contrast, surface expression of CD7 in AML was heterogeneous, with expression in blasts of patients that were clinically defined as CD7-positive varying between 50% and 98% (Figure 3A, $n = 6$). Co-culture of T-ALL cells with K12 CAR-T cells prominently induced apoptosis in all primary T-ALL cells, reaching up to ∼95%, whereas treatment with CD19 CAR-T cells did not significantly induce apoptosis compared to primary T-ALL monocultures (Figures 3B and 3C). Further, residual live tumor cell count was strongly reduced compared to the total number of seeded cells by K12 CAR-T treatment (Figure 3D, $p < 0.001$). In contrast, no difference in the number of residual live tumor cells was detected in CD19 CAR-T-treated and T-ALL monocultures (Figure 3D). K12 CAR-T cells also secreted higher levels of IFN-γ in the supernatant ranging from 14 to 23 ng/mL, whereas CD19 CAR-T treatment did not significantly induce IFN-γ secretion (Figure 3E). K12 CAR-T cells also induced apoptosis in patient-derived AML cells to ∼80% apoptosis (Figure 3F) and triggered significant secretion of IFN-γ compared to CD19 CAR-T-treated and AML monocultures (Figure 3G, $p < 0.05$). Indeed, K12 CAR-T cell treatment eliminated all CD7-positive (CTV-negative) blasts (Figure 3H), but residual viable CD7-negative AML blasts were detected after treatment (Figure 3H). In conclusion, K12 CAR-T cells specifically triggered apoptotic elimination of CD7-positive T-ALL/AML cells. However, for AML, residual CD7-negative blasts survived the treatment that could contribute to treatment failure and relapse.

K12 CAR-T cells protected against systemic T-ALL *in vivo*

The ability of K12 CAR-T cells to treat T-ALL *in vivo* was evaluated in sub-lethally irradiated NSG mice by intravenously injecting Jurkat

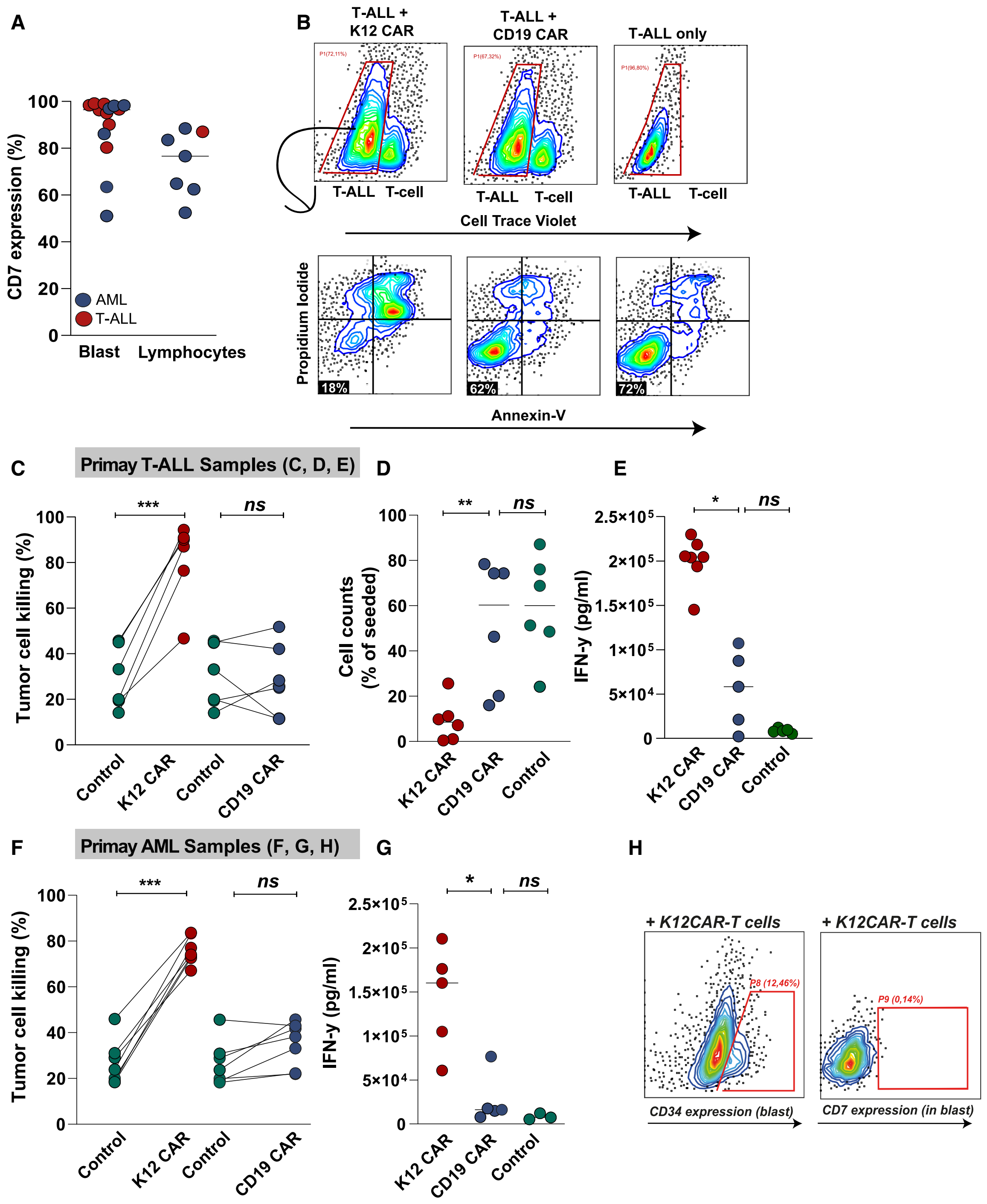

Figure 3. K12 CAR-T cell activity toward primary CD7-positive T-ALL- and AML-patient-derived samples

(A) Percentage of CD7 expression in T-ALL- and AML-patient-derived samples, divided into blasts and lymphocytes, as measured by flow cytometry. (B) Gating strategy for assessing cytotoxicity by K12 and CD19 CAR-T cells to target cells. (C) Percentage of cell death of patient-derived T-ALL cells after treatment with K12 CAR-T cells and CD19 CAR-T cells for 48 h. (D) Quantification of cell counts of patient-derived T-ALL cells after treatment with K12 CAR-T cells and CD19 CAR-T cells. (E) IFN-γ secretion by CAR-T cells after 48 h treatment of patient-derived T-ALL cells. (F) Percentage of cell death in patient-derived AML cells after 48 h treatment with K12 CAR-T cells and CD19 CAR-T cells. (G) IFN-γ secretion by CAR-T cells after 48 h treatment of patient-derived AML cells. (H) Illustrative flow cytometry plots of residual (CTV-negative) AML cells after K12 CAR T cell treatment, including CD7 expression into the blast (CD34 positive) population. ***$p < 0.001$, **$p < 0.01$, and *$p < 0.05$.

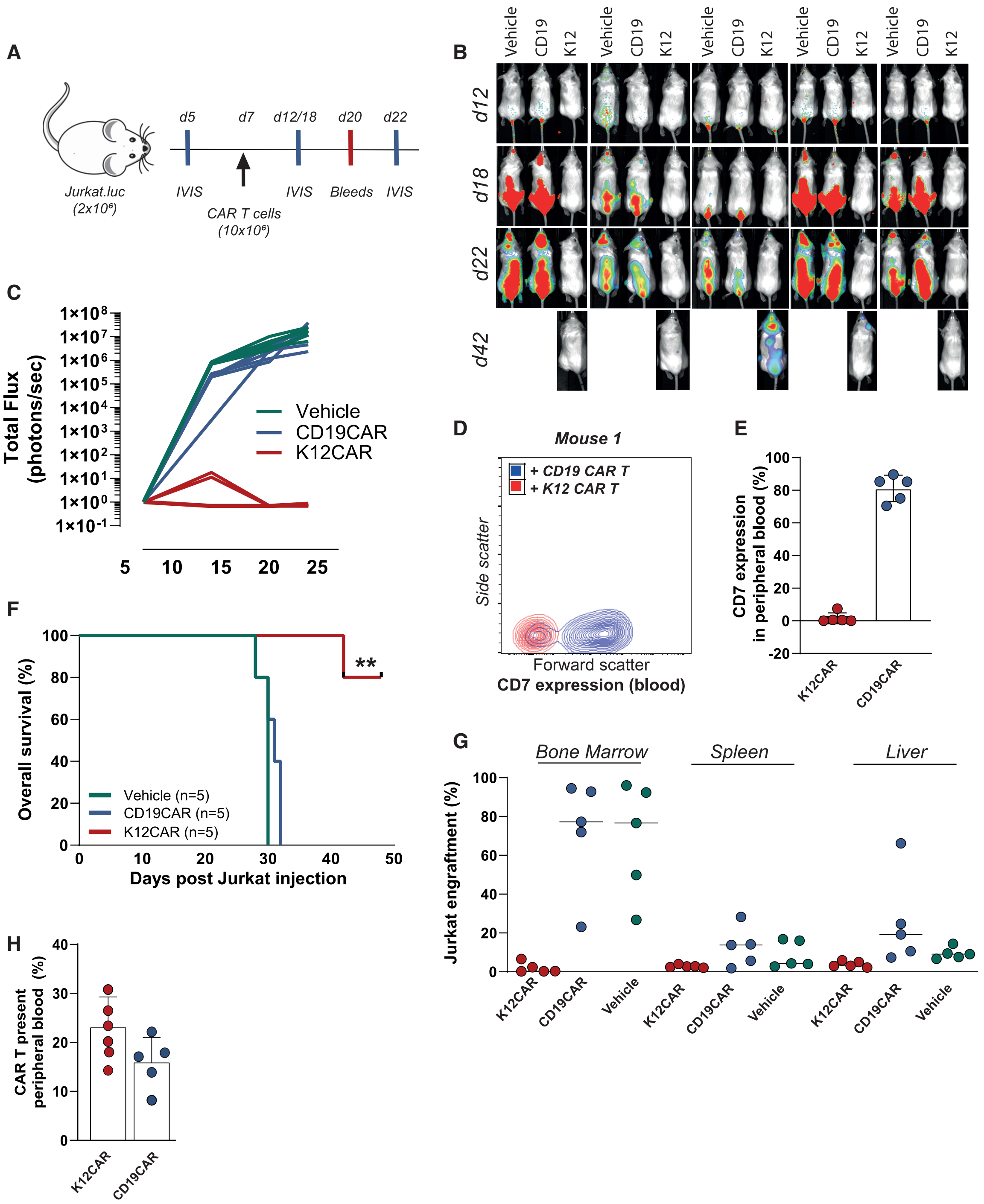

Figure 4. K12 CAR-T cells eliminated Jurkat cells in a murine xenograft model

(A) Schematic overview of experimental timeline of the *in vivo* Jurkat xenograft model. (B) Tumor load monitored at different time points using bioluminescence imaging. (C) Quantification of the total radiance in the images presented in (B). (D and E) CD7 expression in the peripheral blood of mice. (F) Kaplan-Meier survival curve for mice treated with vehicle, CD19 CAR T-cells, and K12 CAR T-cells. (G) Jurkat engraftment in bone marrow, spleen, and liver after mice was sacrificed. (H) CAR-T cell presence in the peripheral blood. ***p* < 0.01.

cells expressing firefly luciferase followed by treatment with K12 CAR-T cells, CD19 CAR-T cells, or vehicle control (Figure 4A). Systemic leukemia development, as evaluated by bioluminescence, was detected on day 12 in the control and CD19 CAR-T-cell-treated groups, whereas no systemic leukemia was detected in K12 CAR-T-cell-treated mice (Figures 4B and 4C). In line with this, no CD7-positive cells were detected in peripheral blood from K12 CAR-T-cell-treated mice, whereas control mice had a significant percentage of CD7-positive cells in the blood (Figures 4D and 4E). Treatment with K12 CAR-T cells significantly prolonged OS, with no leukemia being detected in three out of five mice treated with K12 CAR-T cells at the endpoint of the study (>42 days) (Figures 4B, 4C, and 4F). In contrast, OS of vehicle and CD19 CAR-T-cell-treated mice was only 29 and 31 days, respectively (Figure 4F). Notably, both K12 and CD19 CAR-T cells were present in peripheral blood at day 20, at around 20% of the total cell count (Figure 4H). Furthermore, Jurkat engraftment was absent in the bone marrow, spleen, and liver of four out of the five mice treated with K12 CAR-T cells, with one mouse exhibiting approximately 8% engraftment of CD7-positive Jurkat cells in the bone marrow on day 42. In contrast, engraftment of Jurkat in the bone marrow of CD19 CAR-T-cell-treated and vehicle control mice reached up to 95%, yielding a white cancerous bone marrow and enlarged spleen (Figure S3A). In line with this, spleens in the control groups were also heavier in weight compared to the K12 CAR-T cell group (60 vs. 30 gram) (Figure S3B). The body weight of K12 CAR-T-cell-treated mice was stable over time, whereas body weight of mice treated with vehicle control or CD19 CAR-T cells started to drop from day 14 (Figure S3C). Taken together, K12 CAR-T cells selectively eliminated Jurkat cells *in vivo*.

DISCUSSION

In the current study, we demonstrated that K12 CAR-T cells are more effective at triggering downstream NFAT signaling in a Jurkat-luciferase model system and the K12 CAR-T cells produced more IFN-γ upon CD7-specific triggering than scFvCD7 CAR-T cells. K12 CAR-T cells generated from CD7KO T cells effectively eliminated CD7-positive tumor cell lines as well as T-ALL and CD7-positive AML-patient-derived samples *in vitro*. K12 CAR-T cells also had prominent anti-leukemic activity in an intravenously injected leukemia mouse model, with no leukemia detected in three out of the five mice. Taken together, K12 CAR-T cells exhibited better activity *in vitro* than scFvCD7 CAR-T cells and may hold promise for the treatment of CD7-positive relapsed/refractory T cell leukemia/lymphoma and AML. In our study, we directly compared a ligand-based CAR with an scFv-based CAR in terms of signaling output, with the ligand-based K12 CAR-T yielding higher activity and longer persistence. Based on our data, the ligand-based K12 CAR-T cells were more effective compared to scFvCD7 CAR-T cells to target CD7-positive malignancies. In line with this, various other ligand-based CAR-T cells are being introduced, including granulocyte-monocyte colony stimulating factor (GM-CSF) receptor (GMR) CAR-T cells and FLT3L CAR-T cells in AML.[37,38] The latter had specific cytotoxicity against FLT3+ leukemia cell lines and primary patient-derived AML *in vitro* and significantly prolonged survival of AML-bearing mice *in vivo*.[38] Of note, the difference observed between scFvCD7 and K12 CAR-T cells might perhaps be explained by the different kinetics of binding and synapse formation. In general, scFv have a higher affinity for their target than a natural ligand. This lower affinity in natural interactions is essential for reversible binding, allowing for dynamic regulation of receptor signaling in physiological conditions. In this context, the affinity of scFvCD7 is ∼2 nM[39] and of K12 for CD7 around 10 to 38 nM.[27,28] Low-affinity CAR-T cells have shown promising results in literature in terms of improved cytotoxicity, enhanced expansion and persistence, and reduction of T cell exhaustion.[40–42] The distinct binding kinetics may explain the prolonged persistence of K12CAR-T cells over scFvCD7 CAR-T cells. This all suggests that a ligand-based CAR may be an effective alternative to scFv-based CAR-T cells. Further, scFv CARs can aggregate and trigger tonic signaling, which promotes T cell exhaustion and adversely affects *in vivo* efficacy.[21,26] Thus, a ligand-based CAR such as the K12 CAR-T described here is an attractive alternative.

The activity profile of the K12 CAR-T reported here is in line with a previous published study, in which a second-generation ligand-based K12 (SECTM1) CAR-T cell eliminated cancer cells with high CD7 expression and secreted IFN-γ. Notably, although tumor growth in mice treated with the second-generation K12 CAR-T cells was delayed, cure was not achieved.[12] With the third-generation K12 CAR-T treatment reported here, cure was achieved in three out of five mice. Although models are different, with different cell lines being used, these data may indicate that for T-ALL a third-generation CAR-T may be appropriate. In this respect, a clinical study of third- versus second-generation CD19 CAR-T cells for B cell non-Hodgkin lymphoma identified better persistence and up to 40-fold higher expansion with the third-generation CD19 CAR-T cells.[33] In line with this, ∼20% of peripheral blood cells were K12 CAR-T cells at day 20 of treatment, suggesting this third-generation CAR has good persistence. However, not all clinical evaluations demonstrated this advantage,[43] thus this should be defined for each CAR in a direct comparative study.

Although K12 CAR-T cells had similar cytotoxicity toward CD7-positive tumor cell lines as scFvCD7 CAR-T cells in short-term cytotoxicity assays, K12 CAR-T cells were more prominently activated and secreted more IFN-γ and had a prolonged persistence *in vitro* compared to scFvCD7 CAR-T cells. Prolonged CAR-T cell persistence has been linked to improved clinical outcomes and lower relapse rates in large B cell lymphoma (LCBL), acute lymphoblastic leukemia (ALL), and chronic lymphocytic leukemia (CLL).[44,45] Thus, K12 CAR-T cells might have therapeutic advantages over the antibody-based CAR construct evaluated here.

In literature, different approaches have been reported to manufacture CD7-targeted CAR-T cells, most notably via knockout of CD7 prior to CAR transduction or via a so-called natural selection (NS) method, in which the CAR construct is directly incorporated into the T cells without any CD7 modification or masking beforehand.

In a comparative study of different CD7-targeted manufacturing approaches (NS, CD7-negative sorting, and CD7 KO),[12] the percentage of naive T cells (T_N, CD45RA+, CD62L+) was higher in the CD7KO-CAR-T cells than for NSCD7 CARs. CD7KO cells had better growth kinetics, with a 45-fold expansion compared to a 12-fold expansion of NS7CAR in a 2-week culture assay. However, in the NSCD7 CAR-T cell group 92.4% of cells expressed the CD7 CAR, whereas only 34% did in CD7KO CAR-T cells. The latter finding contrasts with our data, in which >90% of cells expressed the CAR construct upon CD7KO. For the clinical study, 20 eligible patients were infused with NS7CAR-T cells, with 19/20 patients achieving minimal residual disease (MRD)-negative complete remission (CR) in the bone marrow (BM) and an 18-month OS rate of 75%.[12] Although CR and OS rates achieved using NS CAR-T cells were promising, the infused product had a relatively exhausted phenotype comprising ~64% effector memory and 7% terminally differentiated cells. Notably, for CD19 CAR T cell therapy, the percentage of naive and central memory T cells positively correlates with survival.[46]

To improve the fitness of the CAR-T product, the use of allogeneic T cells instead of autologous T cells can be explored, although for CD19 CAR-T therapy the use of autologous cells generally outperformed allogeneic CAR-T cells, particularly in preventing relapse.[47] Interestingly, for CD7 CAR-T cells, patients who were treated with allogeneic CD7 CAR-T cells had a higher CR-rate, higher persistence at month 2, and a lower relapse rate compared to patients treated with autologous CAR-T cells.[11] However, use of allogeneic CAR-T cells introduces the risk of graft-versus-host disease (GvHD).[47,48] Thus, for the further development of K12 CAR-T cells, the choice of donor may be disease-related, e.g., allogeneic for T-ALL and autologous for AML. Importantly, as patients with T cell malignancies have abnormal/malignant T cells in peripheral blood, it is imperative to ensure that no malignant leukemic CAR is generated during manufacturing when CAR-Ts are manufactured from autologous T cells. In clinical studies, exclusion of abnormal/malignant T cells for CAR T cell manufacturing has been implemented, e.g., for patients with CD3-negative blasts.[12,49]

CD7 is highly expressed in almost all T-ALL and T cell lymphoma patients, with malignant cells within a patient also being uniformly positive for CD7. In contrast, CD7 is detected only in malignant cells in approximately 30% of AML patients, with not all AML blasts within a patient always being positive for CD7. Indeed, CD7 expression ranges from 50% to 98% in AML patients in our dataset. Thus, treatment with a CD7-targeted CAR-T could lead to continued development of CD7-negative malignancy. Indeed, during *in vitro* treatment as described here, CD7-negative malignant blasts survived and, in a phase I clinical trial, two of eight AML patients relapsed with CD7-negative leukemia after initially achieving a complete remission with CD7 CAR T cell therapy. Nevertheless, CD7-targeted CAR-T treatment might be of interest also in those patients with a percentage of CD7-negative blasts, since high expression of CD7 in AML patients is associated with more progressive disease and poor prognosis to standard therapy. As such, drug resistance may reside in the CD7-expressing malignant clone.[50,51] Thus, CD7 treatment could restore drug sensitivity and pave the way for combination or sequential treatment with chemotherapy.

In conclusion, K12 CAR-T cells generated from CD7KO-T cells significantly outperformed scFvCD7 CAR-T cells in terms of T cell activation, cytokine secretion, and persistence. Further, K12 CAR-T cells effectively eliminated CD7-positive tumor cell lines as well as primary patient-derived CD7-positive T-ALL and AML *in vitro* and had prominent anti-leukemic activity in an intravenously (i.v.) injected leukemia xenograft mouse model, with complete remission in 60% of mice. Taken together, this third-generation K12 CAR-T cell has prominent anticancer activity and may be of interest for relapsed/refractory patients with CD7-positive T cell leukemia/lymphoma and AML.

MATERIAL AND METHODS

Culture conditions of cell lines and patient-derived samples

The leukemia cell lines Jurkat (E6-1), CCRF-CEM, Molt-4, the histiocytic lymphoma cell line U-937, the cutaneous T cell lymphoma cell line Hut78, and the Burkitt lymphoma cell line Ramos were obtained from the American Type Culture Collection (ATCC, Manassas, VA). Ramos.CD7 was generated as described before.[52] Cell lines were maintained in RPMI 1640 (Thermo Fisher Scientific, Waltham, MA) supplemented with 10% fetal calf serum (FCS) (Thermo Fisher Scientific Waltham, MA, USA) and 1% penicillin-streptomycin (Sigma-Aldrich, St. Louis, MO, USA) according to ATCC recommendations. The transduced cell line, Ramos.CD7, was cultured in the presence of 500 μg/mL Hygromycin B (Thermo Fisher Scientific, Waltham, MA, USA).

Peripheral blood (PB) and bone marrow (BM) samples of T-ALL and AML patients were obtained after informed consent and protocol approval by the Medical Ethical committee of the UMCG in accordance with the Declaration of Helsinki (protocol code NL43844.042.13, 6 January 2014). Mononuclear cells (MNCs) were isolated by Ficoll separation (Lymphoprep, Bernburg, Germany) and cryopreserved. Cryovials of patients material were thawed in pre-warmed newborn calf serum (NCS, Thermo Fisher Scientific, Waltham, MA), spun down 5 min at 450 g. Thereafter, the pellet was re-suspended in pre-warmed NCS mix (4 μM magnesium sulfate (Sigma-Aldrich, St. Louis, MO), 20 U/mL DNase (Roche, Basel, Switzerland), and 5 U/mL Heparin (Pharmacy of the UMCG, the Netherlands)) and incubated at 37°C for 15 min and harvested (5 min, 450 g). T-ALL/AML patient cells were cultured in AlphaMEM (Sigma-Aldrich, St. Louis, MO, USA) supplemented with 20% FCS, granulocyte colony-stimulating factor (G-CSF), interleukin-3 and -7 (IL-3, IL-7) (Immunotools, Friesoythe, Germany), and N-plate (Pharmacy of the UMCG, the Netherlands).

CAR design and lentiviral packaging

A CAR containing the extracellular domain of K12 was gene-synthesized (Genscript) into a backbone comprising the human CD8 hinge

and transmembrane domain, the 4-1BB and CD28 costimulatory domains, and the CD3ζ signaling domain in the lentiviral vector pRRL, yielding pRRL-K12 CAR-iGFP. Similar constructs were generated for CD19 (pRRL-scFv-CD19-CAR-iGFP) and CD7 (pRRL-scFv-CD7-CAR-iGFP). Non-transduced (NT) T cells and T cells transduced with the CD19 CAR were used as controls throughout the experiments. Lentivirus production was performed by transfection of HEK-293T cells with transfer plasmid pRRL K12-CAR or pRRL scFv3A1F-CAR and packaging plasmids pMD2, pMDL, and pRSV (Genscript, Rijswijk, the Netherlands) at a molar ratio of 6:1:4:1. HEK-293T cells were seeded 18–24 h prior to transfection at a seeding density of 0.52 × 105 cells/cm^2 in DMEM (Lonza, Thermo Fisher Scientific, Waltham, MA) supplemented with 10% FCS (Thermo Scientific Waltham, MA, USA). Media was exchanged 1–2 h prior to transfection with transfection media (2% FCS containing DMEM) (to a total of 9 mL for T75 flask). The transfection mixture was prepared by mixing the plasmids in the ratio described earlier in serum-free DMEM (to 10% of the final volume of each transfection well/flask) and briefly vortexed. Subsequently PEI MAX (Polysciences, Hirschberg an der Bergstrasse, Germany) was added at a concentration of 0.021 μg/mL (PEI MAX/DNA complex) and then mixed by pipetting. PEI MAX/DNA complex was incubated for 20 min at room temperature (RT) and then added dropwise to the cells. Virus was harvested 48 h after transfection (450 g, 5 min), concentrated with Amicon Ultra-15 Centrifugal Filter Unit (Merck, Darmstadt, Germany) (4,000 g, 20 min), and stored in RPMI 1640 (Lonza, Thermo Fisher Scientific, Waltham, MA) at −80°C in aliquots.

Generation of CD7 KO T cells and transduction of the CAR construct

To minimalize fratricidal effects toward Jurkat$^{NFAT\text{-}luc}$ and T cells upon CAR expression, CD7 KO was performed on isolated CD4/CD8-selected T cells prior to lentiviral transduction. CD4/CD8 T cells were isolated from PBMCs obtained from buffy coats according to manufacturer's protocol using CD4 and CD8 MicroBeads (Miltenyi Biotec, Leiden, the Netherlands) using the MACS separator (Miltenyi Biotec, Leiden, the Netherlands). CD7 KO was performed by electroporation using CRISPR Cas9 technology: Alt-R CRISPR-Cas9 tracrRNA 100 μM, Alt-R S. P. HiFi Cas9 Nuclease V3 10 mg/mL, crRNA guide: ATGCTCGGACGCCCCA CCAA, 100 μM (Integrated DNA Technologies [IDT], Iowa, USA), and P2 primary cell 4D-nucleofector X kit (Lonza, Thermo Fisher Scientific, Waltham, MA). For KO of 10 × 10^6 CD4/CD8-selected T cells, 1.5 μL of the crRNA guide and 1.5 μL of TracRNA were mixed by vortex, incubated at 95°C for 5 min and 10 min at RT and then mixed with 12 μL of duplex Buffer (TRAC RNA MIX). Twelve microliters of the TRAC RNA mix was then mixed with 3.2 μL of Duplex Buffer and 0.8 μL of Cas9 (RNP MIX) and incubated for 10 min at RT. Cells were washed with PBS, all the supernatant was removed, and cells were resuspended in 100 μL of nucleofection solution (82 μL P2 cell solution + 18 μL supplement), mixed with 15 μL of the RNP mix and incubated for 2 min at RT. Then the mix was transferred to electroporation cuvettes and electroporated in the 4D-Nucleofector (Lonza, Thermo Fisher Scientific, Waltham, MA). Cells were then incubated in RPMI 1640 supplemented with 20% FCS and 6,000 IU/mL IL-2 (Pharmacy of the UMCG) for 72 h to allow for downregulation of CD7 expression. T cells were activated using CD3/CD28 Dynabeads Human T-activator (Thermo Scientific, Waltham, MA, USA) 24 h prior to lentiviral transduction. Specifically, T cells were seeded at a density of 1 × 10^6/mL in RPMI 1640 supplemented with 20% FCS, 6,000 IU/mL IL-2 and transduced with lentivirus encoding K12 CAR, scFvCD7 CAR, or CD19 CAR. Seventy-two hours after transduction, T cells were washed with PBS supplemented with 2% FCS, and transduction efficiency was measured using a CytoFLEX flow cytometer (Beckman Coulter Brea, CA, USA).

Surface Markers and CAR Detection

Fluorescein isothiocyanate (FITC) or allophycocyanin (APC)-conjugated CD3 (SK7), CD5 (UCHT2), CD45 (HI30), and CD7-6B7 (Biolegend, California, USA) were used to detect T cell surface markers. CAR surface expression was detected with biotinylated recombinant human CD7 (Biolegend, California, USA) followed by Alexa Fluor 647-conjugated streptavidin. All flow cytometry data were obtained on the CytoFLEX flow cytometer (Beckman Coulter Brea, CA, USA) using accessory software.

Cytotoxicity of CAR-T cells and cytokine secretion

For cytotoxicity, CAR-T cells were stained with Cell Trace Violet (CTV) (Thermo Scientific Waltham, MA, USA) and mixed with tumor cells (cell lines or primary patient-derived samples) at various E:T ratios in RPMI 1640 supplemented with 10% FCS. After 48 h, cytotoxicity was evaluated by staining of cultures with Annexin V (AnnV, Biolegend, California, USA) and propidium iodide (PI, Biolegend, California, USA) according to manufacturer's recommendations and analyzed by flow cytometry. First, CTV-negative target cells were gated, whereafter AnnV/PI expression was determined. Supernatant of the co-culture was harvested and stored at −20°C for later assessment of IFN-γ levels using IFN-γ ELISA according to manufacturer's protocol (ImmunoTools, Friesoythe, Germany). To determine CAR-T cell activation, K12-, scFvCD7-,and CD19 CAR-T cells were incubated for 24 h with Dynabeads M-280 Streptavidin (Thermo Fisher Scientific Waltham, MA, USA) alone or loaded with Biotinylated Recombinant Human CD7 (Biolegend, California, USA) or CD19 CAR detection reagent (Miltenyi Biotec, Leiden, the Netherlands) at an E:T ratio of 1:10. Supernatant was harvested after 24 h, whereupon IFN-γ levels were determined using a Simple Plex Human IFN-gamma (3rd Gen) Cartridge in the ELLA (Bio-Techne, Minneapolis, USA). The level of IFN-γ in the non-loaded bead condition was subtracted from the other conditions to yield the ΔIFN-γ levels.

Luminescence-based tumor-rechallenge assay

To evaluate the persisting cytotoxicity capacity after several tumor re-challenges, K12 CAR-T cells were co-cultured with the adherent luciferase-expressing OVCAR-3.CD7-mCherry. These cells were pre-seeded 24 h prior to the assay as 10 × 10^5 cells/well in 100 μL

RPMI 10% FCS in 96-well plates. After that, 100 μL of K12 CAR-T cells were added according to E:T 1:1. After that, complete supernatant (containing the K12 CAR-T cells) was transferred to a new 96-well plate, whereas 100 μL of PBS was added to the assay plate (containing the OVCAR-3-mCherry cells). First, the remaining viable OVCAR-3.CD7-mCherry cells were visualized with fluorescent microscopy EVOS Cell Imaging System (EVOS-FL, Thermo Scientific, Waltham, MA, USA). Afterward, Bio-Glo luciferase assay system (Promega, kit #G7941) was added to the assay plate according to the manufacture's recommendation and incubated for 15 min at RT in the dark. Immediately after, a luminescence readout was performed using a luminescence reader (Synergy, BioTek, Winooski, VT, USA). Relative light unit (RLU) was recorded and analyzed by Gen5 data analysis software (BioTek). Finally, the 200 μL assay supernatant (containing the K12 CAR-T cells) that had been previously collected was centrifuged for 5 min at 300 g, and supernatant was removed, leaving a residual volume of 100 μL. This volume (containing the K12 CAR-T cells) was then transferred to a new 96-well plate containing previously seeded OVCAR-3-mCherry cell lines as previously described. This cycle was repeated for six times.

In vivo xenograft model

Healthy 8-week-old female NSG (NOD.Cg-Prkdcscid Il2rgtm1Wjl/SzJ) mice were purchased from the Centrale Animal breeding facility of the UMCG. Mouse experiments were performed in accordance with national and institutional guidelines, and all experiments were approved by the Institutional Animal Care and Use Committee of the University of Groningen. Prior to transplantation, mice were sublethally irradiated with a dose of 1.0 Gy. Mice received a single intravenous injection of 2×10^6 Jurkat.luciferase cells. Bioluminescence was measured on days 5, 7, 12, 18, 22, and 42 post-injection using the IVIS spectrum. Hereto, mice were injected with 100 μg luciferin substrate (GoldBio, Missouri, USA) in the retro-orbital plexus. At day 7, mice were i.v. injected with vehicle control, CD19 CAR-T cells, or K12 CAR-T cells via the tail vein. CAR-T cell presence and Jurkat.luciferase engraftment was determined by the detection of human CD45, CD3, and CD7 (Biolegend, California, USA) in peripheral blood on day 20. Bodyweight was measured three times per week for the duration of the experiment. At termination, the mice were humanely euthanized via cervical dislocation under isoflurane anesthesia. Blood, spleen, liver, bone marrow, and any tumors were collected, whereafter CD45, CD3, and CD7 expression levels were detected using CytoFLEX flow cytometer (Beckman Coulter Brea, CA, USA) with accessory software.

Statistical analysis

All statistical analyses were performed using GraphPad Prism software (GraphPad Inc, CA, USA) and represented as the mean ± standard deviation (SD). Statistical significance between two groups was calculated using a two-tailed unpaired Student's t test. Correlation between CD7 expression of targeted cells and IFN-γ secretion was analyzed using Pearson correlation. Statistical significance in Kaplan-Meier survival curves was assessed with the Mantel-Cox log rank test. p values are indicated as $^{****}p < 0.0001$, $^{***}p < 0.001$, $^{**}p < 0.01$, and $^{*}p < 0.05$.

DATA AVAILABILITY

All data generated or analyzed during this study are included in this published article (and its supplementary information files).

ACKNOWLEDGMENTS

We would like to acknowledge the support from the Just Transition Fund (JTF) grant Excellent and EU Marie Curie Doctoral Network InnoCAR-T (GA101072861).

Peripheral blood (PB) and bone marrow (BM) samples of T-ALL and AML patients were studied after informed consent and protocol approval by the Medical Ethical committee of the UMCG in accordance with the Declaration of Helsinki (protocol code NL43844.042.13, 6 January 2014).

AUTHOR CONTRIBUTIONS

E.B., N.V., and M.G.C. conceived the experiments. E.B., N.V., and M.G.C. designed the experiments. N.V., M.G.C., and M.G.P. performed the experiments. H.J.L. contributed reagents and material. N.V. and M.G.C. analyzed the data. N.V., E.B., and M.G.C. wrote the manuscript. E.B., N.V., M.G.C., J.A.F., L.R., M.G.P., H.J.L., J.J.S., T.V.M., and G.H. reviewed and edited the manuscript.

DECLARATION OF INTERESTS

The authors declare no competing interests.

SUPPLEMENTAL INFORMATION

Supplemental information can be found online at https://doi.org/10.1016/j.omton.2025.200988.

REFERENCES

1. Hu, Y., Zhou, Y., Zhang, M., Zhao, H., Wei, G., Ge, W., Cui, Q., Mu, Q., Chen, G., Han, L., et al. (2022). Genetically modified CD7-targeting allogeneic CAR-T cell therapy with enhanced efficacy for relapsed/refractory CD7-positive hematological malignancies: a phase I clinical study. Cell Res. *32*, 995–1007. https://doi.org/10.1038/s41422-022-00721-y.
2. Scherer, L.D., Brenner, M.K., and Mamonkin, M. (2019). Chimeric Antigen Receptors for T-Cell Malignancies. Front. Oncol. *9*, 126. https://doi.org/10.3389/fonc.2019.00126.
3. Pan, J., Tan, Y., Deng, B., Ling, Z., Xu, J., Duan, J., Wang, Z., Wang, K., and Hu, G. (2023). S116: DONOR-DERIVED CD5 CAR T CELLS FOR T-CELL ACUTE LYMPHOBLASTIC LEUKEMIA. Hemasphere *7*, e3515785. https://doi.org/10.1097/01.HS9.0000967376.35157.85.
4. Feng, J., Xu, H., Cinquina, A., Wu, Z., Chen, Q., Zhang, P., Wang, X., Shan, H., Xu, L., Zhang, Q., et al. (2021). Treatment of Aggressive T Cell Lymphoblastic Lymphoma/leukemia Using Anti-CD5 CAR T Cells. Stem Cell Rev. Rep. *17*, 652–661. https://doi.org/10.1007/s12015-020-10092-9.
5. Patel, J., Gao, X., and Wang, H. (2023). An Update on Clinical Trials and Potential Therapeutic Strategies in T-Cell Acute Lymphoblastic Leukemia. Int. J. Mol. Sci. *24*, 7201. https://doi.org/10.3390/ijms24087201.
6. Ramos, C.A., Grover, N.S., Beaven, A.W., Lulla, P.D., Wu, M.-F., Ivanova, A., Wang, T., Shea, T.C., Rooney, C.M., Dittus, C., et al. (2020). Anti-CD30 CAR-T Cell Therapy in Relapsed and Refractory Hodgkin Lymphoma. J. Clin. Oncol. *38*, 3794–3804. https://doi.org/10.1200/JCO.20.01342.
7. Ramos, C.A., Bilgi, M., Gerken, C., Dakhova, O., Mei, Z., Wu, M.-F., Grilley, B., Gee, A.P., Rooney, C.M., Dotti, G., et al. (2019). CD30-Chimeric Antigen Receptor (CAR) T Cells for Therapy of Hodgkin Lymphoma (HL). Biol. Blood Marrow Transplant. *25*, S63. https://doi.org/10.1016/j.bbmt.2018.12.145.
8. Shi, J., Zhang, Z., Cen, H., Wu, H., Zhang, S., Liu, J., Leng, Y., Ren, A., Liu, X., Zhang, Z., et al. (2021). CAR T cells targeting CD99 as an approach to eradicate T-cell acute lymphoblastic leukemia without normal blood cells toxicity. J. Hematol. Oncol. *14*, 162. https://doi.org/10.1186/s13045-021-01178-z.

9. Zhao, L., Li, C., Zuo, S., Han, Y., Deng, B., Ling, Z., Zhang, Y., Peng, S., Xu, J., Duan, J., et al. (2025). Autologous CD7 CAR-T cells generated without T cell pre-selection in pediatric patients with relapsed/refractory T-ALL: A phase I trial. Mol. Ther. *33*, 2753–2767. https://doi.org/10.1016/j.ymthe.2024.09.006.

10. Wang, X., Li, S., Gao, L., Yuan, Z., Wu, K., Liu, L., Luo, L., Liu, Y., Zhang, C., Liu, J., et al. (2020). Abstract CT052: Clinical safety and efficacy study of TruUCAR™ GC027: The first-in-human, universal CAR-T therapy for adult relapsed/refractory T-cell acute lymphoblastic leukemia (r/r T-ALL). Cancer Res. *80*, CT052. https://doi.org/10.1158/1538-7445.AM2020-CT052.

11. Zhang, Y., Li, C., Du, M., Jiang, H., Luo, W., Tang, L., Kang, Y., Xu, J., Wu, Z., Wang, X., et al. (2023). Allogenic and autologous anti-CD7 CAR-T cell therapies in relapsed or refractory T-cell malignancies. Blood Cancer J. *13*, 61. https://doi.org/10.1038/s41408-023-00822-w.

12. Lu, P., Liu, Y., Yang, J., Zhang, X., Yang, X., Wang, H., Wang, L., Wang, Q., Jin, D., Li, J., and Huang, X. (2022). Naturally selected CD7 CAR-T therapy without genetic manipulations for T-ALL/LBL: first-in-human phase 1 clinical trial. Blood *140*, 321–334. https://doi.org/10.1182/blood.2021014498.

13. Gorczyca, W. (2021). Atlas of Differential Diagnosis in Neoplastic Hematopathology, 4th ed. (CRC Press).

14. Campana, D., Van Dongen, J.J.M., Mehta, A., Coustan-Smith, E., Wolvers-Tettero, I. L.M., Ganeshaguru, K., and Janossy, G. (1991). Stages of T-cell Receptor Protein Expression in T-cell Acute Lymphoblastic Leukemia. Blood *77*, 1546–1554.

15. Bonilla, F.A., Kokron, C.M., Swinton, P., and Geha, R.S. (1997). Targeted gene disruption of murine CD7. Int. Immunol. *9*, 1875–1883.

16. Gomes-Silva, D., Srinivasan, M., Sharma, S., Lee, C.M., Wagner, D.L., Davis, T.H., Rouce, R.H., Bao, G., Brenner, M.K., and Mamonkin, M. (2017). CD7-edited T cells expressing a CD7-specific CAR for the therapy of T-cell malignancies. Blood *130*, 285–296. https://doi.org/10.1182/blood-2017-01-761320.

17. Reinhold, U., and Abken, H. (1997). CD4+ CD7- T cells: a separate subpopulation of memory T cells? J. Clin. Immunol. *17*, 265–271. https://doi.org/10.1023/a:1027318530127.

18. Freiwan, A., Zoine, J.T., Crawford, J.C., Vaidya, A., Schattgen, S.A., Myers, J.A., Patil, S.L., Khanlari, M., Inaba, H., Klco, J.M., et al. (2022). Engineering naturally occurring CD7- T cells for the immunotherapy of hematological malignancies. Blood *140*, 2684–2696. https://doi.org/10.1182/blood.2021015020.

19. Wei, W., Yang, D., Chen, X., Liang, D., Zou, L., and Zhao, X. (2022). Chimeric antigen receptor T-cell therapy for T-ALL and AML. Front. Oncol. *12*, 967754. https://doi.org/10.3389/fonc.2022.967754.

20. Wong, X.F.A., Ng, J., Zheng, S., Ismail, R., Qian, H., Campana, D., and Tan, Y.X. (2022). Development of an Off-the-Shelf Chimeric Antigen Receptor (CAR)-T Cell Therapy for T-Cell Acute Lymphoblastic Leukemia (T-ALL) without Gene Editing. Blood *140*, 2358–2359. https://doi.org/10.1182/blood-2022-165822.

21. Frigault, M.J., Lee, J., Basil, M.C., Carpenito, C., Motohashi, S., Scholler, J., Kawalekar, O.U., Guedan, S., McGettigan, S.E., Posey, A.D., et al. (2015). Identification of chimeric antigen receptors that mediate constitutive or inducible proliferation of T cells. Cancer Immunol. Res. *3*, 356–367. https://doi.org/10.1158/2326-6066.CIR-14-0186.

22. Tan, Y., Shan, L., Zhao, L., Deng, B., Ling, Z., Zhang, Y., Peng, S., Xu, J., Duan, J., Wang, Z., et al. (2023). Long-term follow-up of donor-derived CD7 CAR T-cell therapy in patients with T-cell acute lymphoblastic leukemia. J. Hematol. Oncol. *16*, 34. https://doi.org/10.1186/s13045-023-01427-3.

23. Hu, Y., Zhang, M., Yang, T., Mo, Z., Wei, G., Jing, R., Zhao, H., Chen, R., Zu, C., Gu, T., et al. (2024). Sequential CD7 CAR T-Cell Therapy and Allogeneic HSCT without GVHD Prophylaxis. N. Engl. J. Med. *390*, 1467–1480. https://doi.org/10.1056/nejmoa2313812.

24. Bennett, M.J., Choe, S., and Eisenberg, D. (1994). Domain swapping: entangling alliances between proteins. Proc. Natl. Acad. Sci. USA *91*, 3127–3131. https://doi.org/10.1073/pnas.91.8.3127.

25. Bennett, M.J., Schlunegger, M.P., and Eisenberg, D. (1995). 3D domain swapping: a mechanism for oligomer assembly. Protein Sci. *4*, 2455–2468. https://doi.org/10.1002/pro.5560041202.

26. Long, A.H., Haso, W.M., Shern, J.F., Wanhainen, K.M., Murgai, M., Ingaramo, M., Smith, J.P., Walker, A.J., Kohler, M.E., Venkateshwara, V.R., et al. (2015). 4-1BB costimulation ameliorates T cell exhaustion induced by tonic signaling of chimeric antigen receptors. Nat. Med. *21*, 581–590. https://doi.org/10.1038/nm.3838.

27. Lyman, S.D., Escobar, S., Rousseau, A.-M., Armstrong, A., and Fanslow, W.C. (2000). Identification of CD7 as a Cognate of the Human K12 (SECTM1) Protein. J. Biol. Chem. *275*, 3431–3437.

28. Lam, G.K., Liao, H.-X., Xue, Y., Munir Alam, S., Scearce, R.M., Kaufman, R.E., Sempowski, G.D., and Haynes, B.F. (2005). Expression of the CD7 Ligand K-12 in Human Thymic Epithelial Cells: Regulation by IFN-γ. J. Clin. Immunol. *25*, 41–49. https://doi.org/10.1007/s10875-005-0356-2.

29. Duan, Y., Chen, R., Huang, Y., Meng, X., Chen, J., Liao, C., Tang, Y., Zhou, C., Gao, X., and Sun, J. (2022). Tuning the Ignition of CAR: Optimizing the Affinity of scFv to Improve CAR-T Therapy. Cell Mol. Life Sci. *79*, 14. https://doi.org/10.1007/s00018-021-04089-x.

30. Ghorashian, S., Kramer, A.M., Onuoha, S., Wright, G., Bartram, J., Richardson, R., Albon, S.J., Casanovas-Company, J., Castro, F., Popova, B., et al. (2019). Enhanced CAR T cell expansion and prolonged persistence in pediatric patients with ALL treated with a low-affinity CD19 CAR. Nat. Med. *25*, 1408–1414. https://doi.org/10.1038/s41591-019-0549-5.

31. De Bruyn, M., Wei, Y., Wiersma, V.R., Samplonius, D.F., Klip, H.G., Van Der Zee, A. G.J., Yang, B., Helfrich, W., and Bremer, E. (2011). Cell surface delivery of TRAIL strongly augments the tumoricidal activity of T cells. Clin. Cancer Res. *17*, 5626–5637. https://doi.org/10.1158/1078-0432.CCR-11-0303.

32. Wei, W., Ma, H., Yang, D., Sun, B., Tang, J., Zhu, Y., Chen, X., Huang, X., Liu, J., Hu, Z., et al. (2023). SECTM1-based CAR T cells enriched with CD7-low/negative subsets exhibit efficacy in CD7-positive malignancies. Blood Adv. *7*, 2941–2951. https://doi.org/10.1182/bloodadvances.2022008402.

33. Ramos, C.A., Rouce, R., Robertson, C.S., Reyna, A., Narala, N., Vyas, G., Mehta, B., Zhang, H., Dakhova, O., Carrum, G., et al. (2018). In Vivo Fate and Activity of Second- versus Third-Generation CD19-Specific CAR-T Cells in B Cell Non-Hodgkin's Lymphomas. Mol. Ther. *26*, 2727–2737. https://doi.org/10.1016/j.ymthe.2018.09.009.

34. Ye, J., Jia, Y., Tuhin, I.J., Tan, J., Monty, M.A., Xu, N., Kang, L., Li, M., Lou, X., Zhou, M., et al. (2022). Feasibility study of a novel preparation strategy for anti-CD7 CAR-T cells with a recombinant anti-CD7 blocking antibody. Mol. Ther. Oncolytics *24*, 719–728. https://doi.org/10.1016/j.omto.2022.02.013.

35. Watanabe, N., Mo, F., Zheng, R., Ma, R., Bray, V.C., van Leeuwen, D.G., Sritabal-Ramirez, J., Hu, H., Wang, S., Mehta, B., et al. (2023). Feasibility and preclinical efficacy of CD7-unedited CD7 CAR T cells for T cell malignancies. Mol. Ther. *31*, 24–34. https://doi.org/10.1016/j.ymthe.2022.09.003.

36. Pan, J., Tan, Y., Wang, G., Deng, B., Ling, Z., Song, W., Seery, S., Zhang, Y., Peng, S., Xu, J., et al. (2021). Donor-Derived CD7 Chimeric Antigen Receptor T Cells for T-Cell Acute Lymphoblastic Leukemia: First-in-Human, Phase I Trial. J. Clin. Oncol. *39*, 3340–3351. https://doi.org/10.1200/JCO.21.00389.

37. Sun, L., and Babushok, D.V. (2020). Secondary myelodysplastic syndrome and leukemia in acquired aplastic anemia and paroxysmal nocturnal hemoglobinuria. Blood *136*, 36–49. https://doi.org/10.1182/blood.2019000940.

38. Wang, Y., Xu, Y., Li, S., Liu, J., Xing, Y., Xing, H., Tian, Z., Tang, K., Rao, Q., Wang, M., and Wang, J. (2018). Targeting FLT3 in acute myeloid leukemia using ligand-based chimeric antigen receptor-engineered T cells. J. Hematol. Oncol. *11*, 60. https://doi.org/10.1186/s13045-018-0603-7.

39. Pauza, M.E., Doumbia, S.O., and Pennell, C.A. (1997). Construction and characterization of human CD7-specific single-chain Fv immunotoxins. J. Immunol. *158*, 3259–3269.

40. Michelozzi, I.M., Gomez-Castaneda, E., Pohle, R.V.C., Cardoso Rodriguez, F., Sufi, J., Puigdevall, P., Subramaniyam, M., Wu, S.W., Guvenel, A., Ghorashian, S., et al. (2020). The Enhanced Functionality of Low-Affinity CD19 CAR T Cells Is Associated with Activation Priming and Polyfunctional Cytokine Phenotype. Blood *136*, 52–53. https://doi.org/10.1182/blood-2020-141249.

41. Olson, M.L., Mause, E.R.V., Radhakrishnan, S.V., Brody, J.D., Rapoport, A.P., Welm, A.L., Atanackovic, D., and Luetkens, T. (2022). Low-affinity CAR T cells exhibit reduced trogocytosis, preventing rapid antigen loss, and increasing CAR T cell expansion. Leukemia *36*, 1943–1946. https://doi.org/10.1038/s41375-022-01585-2.

42. Shabaneh, T.B., Stevens, A.R., Stull, S.M., Shimp, K.R., Seaton, B.W., Gad, E.A., Jaeger-Ruckstuhl, C.A., Simon, S., Koehne, A.L., Price, J.P., et al. (2024). Systemically administered low-affinity HER2 CAR T cells mediate antitumor efficacy without toxicity. J. Immunother. Cancer *12*, e008566. https://doi.org/10.1136/jitc-2023-008566.

43. Enblad, G., Karlsson, H., Gammelgård, G., Wenthe, J., Lövgren, T., Amini, R.M., Wikstrom, K.I., Essand, M., Savoldo, B., Hallböök, H., et al. (2018). A Phase I/IIa Trial Using CD19-Targeted Third-Generation CAR T Cells for Lymphoma and Leukemia. Clin. Cancer Res. *24*, 6185–6194. https://doi.org/10.1158/1078-0432.CCR-18-0426.

44. Fürst, D., Neuchel, C., Neagoie, A., Amann, E., Rode, I., Krauss, A., Schrezenmeier, H., Wais, V., Döhner, H., Viardot, A., and Sala, E. (2022). Monitoring the in-Vivo Expansion and Persistence of CAR-T Cells As a Tool to Help Decision Making in Patients with Aggressive B-Cell Lymphoma. Blood *140*, 7527–7528. https://doi.org/10.1182/blood-2022-169212.

45. Li, C., Zou, D., Wang, D., Fang, B., Huang, H., Li, J., Chen, B., Liu, J., Zhang, X., Dong, Y., et al. (2024). Impact of CAR T-Cell Persistence on Clinical Outcomes in Relapsed/Refractory Multiple Myeloma: Insights from the Phase 2 Fumanba-1 Study. Blood *144*, 4761. https://doi.org/10.1182/blood-2024-204355.

46. Popplewell, L., Wang, X., Blanchard, S., Wagner, J., Naranjo, A., Adria, A., Paul, J., Lim, L., Chang, W.-C., Budde, E.E., et al. (2018). CD19-CAR Therapy Using Naive/Memory or Central Memory T Cells Integrated into the Autologous Stem Cell Transplant Regimen for Patients with B-NHL. Blood *132*, 610. https://doi.org/10.1182/blood-2018-99-119650.

47. Song, F., Hu, Y., Zhang, Y., Zhang, M., Yang, T., Wu, W., Huang, S., Xu, H., Chang, A.H., Huang, H., and Wei, G. (2023). Safety and efficacy of autologous and allogeneic humanized CD19-targeted CAR-T cell therapy for patients with relapsed/refractory B-ALL. J. Immunother. Cancer *11*, e005701. https://doi.org/10.1136/jitc-2022-005701.

48. Shahzad, M., Hussain, A., Siddiqui, R.S., Shah, A.Y., Faisal, M.S., Anwar, I., Tun, A. M., Hoffmann, M., Bansal, R., Shune, L., et al. (2021). Comparison of Efficacy and Safety Profile of Allogeneic Versus Autologous CD19 Chimeric Antigen Receptor T Cell Therapy in Hematological Malignancies: A Systematic Review and Meta-Analysis. Blood *138*, 4824. https://doi.org/10.1182/blood-2021-145669.

49. Zhang, M., Chen, D., Fu, X., Meng, H., Nan, F., Sun, Z., Yu, H., Zhang, L., Li, L., Li, X., et al. (2022). Autologous Nanobody-Derived Fratricide-Resistant CD7-CAR T-cell Therapy for Patients with Relapsed and Refractory T-cell Acute Lymphoblastic Leukemia/Lymphoma. Clin. Cancer Res. *28*, 2830–2843. https://doi.org/10.1158/1078-0432.CCR-21-4097.

50. Chang, H., Salma, F., Yi, Q.l., Patterson, B., Brien, B., and Minden, M.D. (2004). Prognostic relevance of immunophenotyping in 379 patients with acute myeloid leukemia. Leuk. Res. *28*, 43–48. https://doi.org/10.1016/s0145-2126(03)00180-2.

51. Chang, H., Yeung, J., Brandwein, J., and Yi, Q.-L. (2007). CD7 expression predicts poor disease free survival and post-remission survival in patients with acute myeloid leukemia and normal karyotype. Leuk. Res. *31*, 157–162. https://doi.org/10.1016/j.leukres.2006.06.001.

52. Bremer, E., Samplonius, D.F., Peipp, M., van Genne, L., Kroesen, B.-J., Fey, G.H., Gramatzki, M., de Leij, L.F.M.H., and Helfrich, W. (2005). Target Cell–Restricted Apoptosis Induction of Acute Leukemic T Cells by a Recombinant Tumor Necrosis Factor–Related Apoptosis-Inducing Ligand Fusion Protein with Specificity for Human CD7. Cancer Res. *65*, 3380–3388. https://doi.org/10.1158/0008-5472.CAN-04-2756.

OMTON, Volume *33*

Supplemental information

K12-ligand-based CAR T cell therapy for CD7-positive T cell malignancies

Nienke Visser, Macarena González-Corrales, Jimena Álvarez-Freile, Maurien G. Pruis, Lena Rockstein, Harm Jan Lourens, Jan Jacob Schuringa, Tom van Meerten, Gerwin Huls, and Edwin Bremer

Figure S1

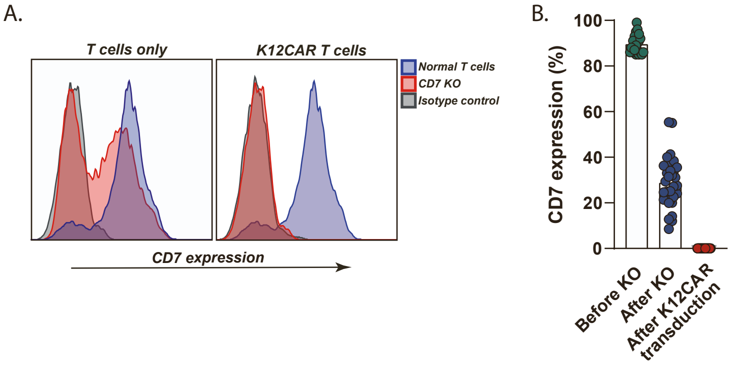

Figure S1 **Fratricidal effect of K12 CAR towards CD7-positive T cells.**
(A-B) CD7 expression in T cells before and after CD7KO and K12 CAR transduction

Figure S2

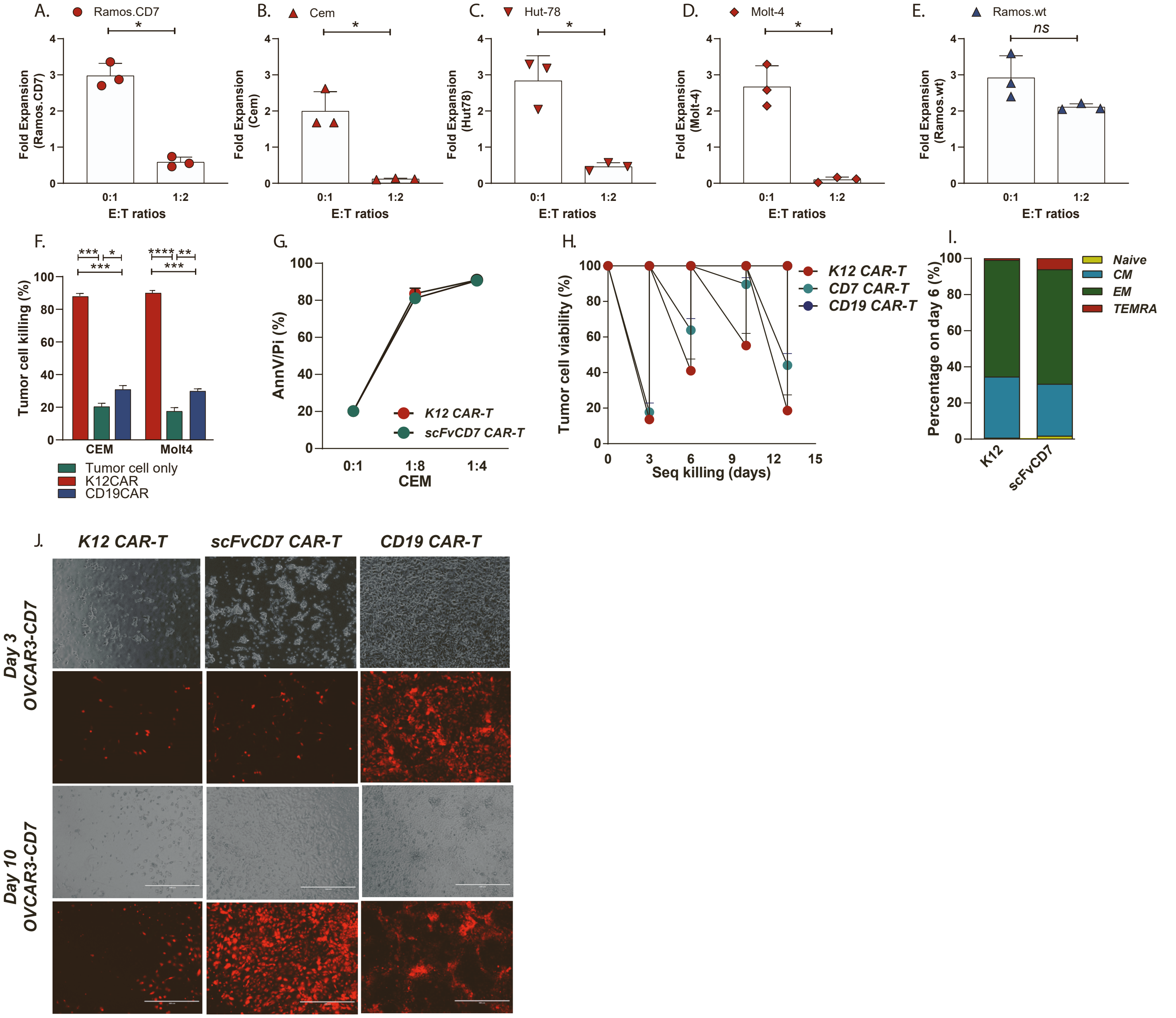

Figure S2 **K12 CAR-T cells selectively eliminated CD7-positive tumor cells.** Remaining tumor cells quantified as Annexin V/PI- negative cells, after treatment with K12 CAR-T cells, for 48h. **(A)** Ramos.CD7, **(B)** CEM, **(C)** Hut-78, **(D)** Molt-4, **(E)** Ramos.wt. **(F)** Cell death of CEM and Molt-4 treated for 48h with K12 CAR or CD19 CAR-T cells. **(G)** 24h killing assay with CEM tumor cell lines, with scFvCD7 CAR-T cell and K12 CAR-T cells. Upon various E:T ratios. **(H)** Sequential killing with OVCAR-3.CD7 and K12, scFv7 and CD19 CAR-T cells. **(I)** T cell subsets of scFvCD7 and K12 CAR T cells after 6 days of manufacturing. **(J)**. Bright-field microscopy pictures of OvCAR-3.CD7-mCherry in co-culture with K12 CAR-T cells, scFvCD7 CAR-T cells and CD19 CAR-T cells, on day 3 and day 10 of the repeated killing assay.
p values are: ****p < 0.0001, ***p < 0.001, **p < 0.01, and *p < 0.05.

Figure S3

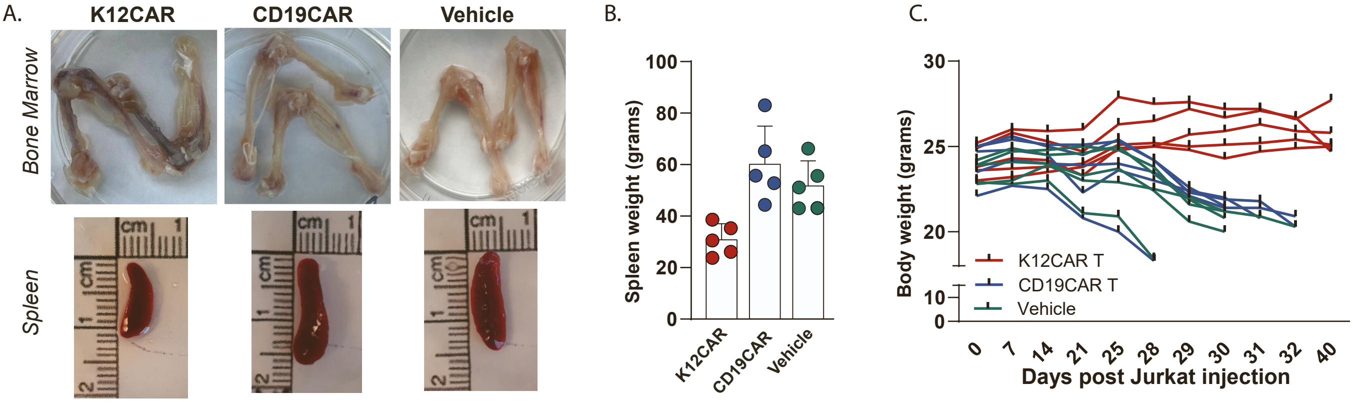

Figure S3 **K12 CAR-T cells eliminated Jurkat cells in a mouse xenograft model**
(A) Representative images of bone marrow and spleen collected post-sacrifice from mice treated with K12, CD19 CAR-T cells, or vehicle control. **(B)** Spleen weight measured post-sacrifice from mice treated with K12, CD19 CAR-T cells, or vehicle control. **(C)** Body weight in grams of the mice treated with K12, CD19 CAR-T cells or vehicle control.
